# Spatial Binding Impairments in Visual Working Memory following Temporal Lobectomy

**DOI:** 10.1523/ENEURO.0278-21.2022

**Published:** 2022-03-08

**Authors:** Mamdouh Fahd Alenazi, Haya Al-Joudi, Faisal Alotaibi, Martyn Bracewell, Neil M. Dundon, Mohammad Zia Ul Haq Katshu, Giovanni d’Avossa

**Affiliations:** 1 School of Psychology, Bangor University, Bangor LL57 2AS, United Kingdom; 2 Department of Neurosciences, King Faisal Specialist Hospital and Research Centre, Riyadh 11211, Saudi Arabia; 3 School of Medical Sciences, Bangor University, Bangor LL57 2AS, United Kingdom; 4 Walton National Health Service Foundation Trust, Liverpool L9 7LJ, United Kingdom; 5 Department of Psychological and Brain Sciences, University of California Santa Barbara, Santa Barbara, CA 93106-9660; 6 Department of Child and Adolescent Psychiatry, Psychotherapy and Psychosomatics, University of Freiburg, Freiburg 79104, Germany; 7 Division of Psychiatry and Applied Psychology, School of Medicine, University of Nottingham, Nottingham NG7 2UH, United Kingdom; 8 Nottinghamshire Healthcare National Health Service Foundation Trust, Nottingham NG3 6AA, United Kingdom

**Keywords:** spatial memory, temporal lobe epilepsy, visual binding, working memory

## Abstract

Disorders of the medial temporal lobe (MTL) adversely affect visual working memory (vWM) performance, including feature binding. It is unclear whether these impairments generalize across visual dimensions or are specifically spatial. To address this issue, we compared performance in two tasks of 13 epilepsy patients, who had undergone a temporal lobectomy, and 15 healthy controls. In the vWM task, participants recalled the color of one of two polygons, previously displayed side by side. At recall, a location or shape probe identified the target. In the perceptual task, participants estimated the centroid of three visible disks. Patients recalled the target color less accurately than healthy controls because they frequently swapped the nontarget with the target color. Moreover, healthy controls and right temporal lobectomy patients made more swap errors following shape than space probes. Left temporal lobectomy patients, showed the opposite pattern of errors instead. Patients and controls performed similarly in the perceptual task. We conclude that left MTL damage impairs spatial binding in vWM, and that this impairment does not reflect a perceptual or attentional deficit.

## Significance Statement

This study examined color recall in temporal lobectomy patients and healthy controls, to determine whether patients show differential impairments binding color and shape versus color and location of memorized objects. Left temporal lobectomy patients were less accurate recalling color, especially when the target object was identified by the location, rather than the shape it had in the initial display. We found no group difference in a task, which required estimating the centroid of three circles, indicating that the memory impairment was not accounted by perceptual or attentional difficulties. Our findings indicate that lateralized medial temporal circuits are crucial for binding visual features to the location where they had appeared, thus ensuring the primacy of space in organizing declarative memories.

## Introduction

The role of the medial temporal lobe (MTL) in episodic memory is well established (Squire, 2009). Despite initial reports of preserved immediate memory span in temporal lobectomy patients (Drachman and Arbit, 1966), later studies found that MTL lesions also engender substantial working memory (WM) deficits (Olton et al., 1979; Holdstock et al., 1995; Hannula et al., 2006). Which WM processes are specifically supported by the MTL is, nevertheless, a matter of ongoing investigations.

An early, seminal model suggested that visual WM (vWM) contains few discrete “slots,” each used to store one and only one object with high fidelity (Luck and Vogel, 1997; Zhang and Luck, 2008). Despite its simplicity, the slot model makes nontrivial predictions. First, the complexity of memorized objects should not affect recall accuracy. Second, recalling feature conjunctions should carry no additional cost over remembering features, since features are stored ipso facto as parts of an object into vWM. Wheeler and Treisman (2002) found, instead, that simple objects were recalled more accurately than complex ones, and that recall accuracy was equalized for displays containing the same number of color features rather than the same number of objects. They concluded that memory limitations reflect feature rather than object-based storage mechanisms. Moreover, observers were worse at detecting changes of feature conjunctions than features, indicating that conjunctions are stored or recalled less efficiently than features. Later studies confirmed that changes in feature conjunctions are poorly detected (Allen et al., 2014), leading to the suggestion that dimensionally specific registers store features, while an “episodic buffer” is dedicated to holding bound object representations in vWM (Baddeley et al., 2011). The need for binding processes follows logically from the alternative model of vWM which proposes that visual features are stored in dimensionally specific, limited resolution stores (Alvarez and Cavanagh, 2004; Smyrnis et al., 2005; Bays et al., 2009). Clearly, if different feature dimensions are stored separately, then a binding process is required to ensure that features belonging to the same object, but different feature dimensions, are identified as such (Wheeler and Treisman, 2002; Smyrnis et al., 2005).

While the idea that conjunctive binding is required to preserve object identity is not unanimously shared (Luck and Vogel, 2013), there are several proposals regarding its nature. Treisman and Zhang (2006) concluded that binding is automatic, established initially by the features’ shared location, but then becomes location independent. Schneegans and Bays (2017) proposed instead that location information is always required, because features from different visual dimensions are stored in separate retinotopic maps.

Investigators examining the neurologic underpinnings of declarative memory largely embrace the idea that space plays a crucial role in indexing declarative memories. Animal and patient studies (Chalfonte et al., 1996; Brown and Aggleton, 2001; Eacott and Gaffan, 2005; Piekema et al., 2006; Ranganath, 2010; Libby et al., 2014) documented a functional parcellation of the MTL with separate structures representing, respectively, the environmental layout, the objects within it, as well as binding the latter to the former. According to this view, space is crucial for recalling events, but not for binding object features. Olson et al. (2006) for example, reported that patients with postanoxic or postencephalitic MTL pathology had impaired object-location binding in a WM task. This impairment was unaccounted by either diminished recognition or spatial memory. In animals, MTL lesions are also followed by dissociated impairments in object recognition and recall of object-location conjunctions, suggesting that feature and spatial binding depend on distinct MTL processes (Meunier et al., 1993; Murray and Mishkin, 1998; Malkova and Mishkin, 2003). Studies in temporal lobe epilepsy (TLE) patients reported deficits in spatial recall, spatial binding and visual recognition (Abrahams et al., 1997; Bohbot et al., 1998; Stepankova et al., 2004); however, it remains unclear whether these impairments should be attributed to diminished spatial precision (Kolarik et al., 2016) or a binding deficit (Zokaei et al., 2019) and whether binding impairments are dimensionally general (Hannula et al., 2006; Pertzov et al., 2013) or specific.

To examine these issues, we tested TLE patients who had undergone temporal lobectomies and healthy controls with two tasks used in a previous investigation of a stroke patient (Dundon et al., 2018). The first requires recalling the color of one of two polygons identified by either a location or a shape probe, thus directly pitting spatial versus nonspatial binding. The second probes participants’ ability to estimate the average position of three visible disks. In this task healthy participants show a pseudo-neglect pattern of leftward static errors (Baud-Bovy and Soechting, 2001), which suggests that centroid estimation is sensitive to attentional biases (Dundon et al., 2018). The centroid task was therefore used to highlight the presence of unilateral neglect, which can follow lesions of the nondominant parahippocampal cortex (Mort et al., 2003) as well as the integrity of spatial perception and attention (Drew et al., 2010).

## Materials and Methods

The aim of the present study was to compare nonspatial and spatial binding performance in TLE patients with medically refractory epilepsy who had undergone temporal lobectomy and healthy controls. Recruitment and testing took place at the Neuropsychology section of the Department of Neurosciences of King Faisal Specialist Hospital and Research Centre in Riyadh, Saudi Arabia. The experimental protocol was approved by the local Institutional Review Board. Participants gave written informed consent before engaging in any experimental procedure.

### Study participants

Over a month period, an opportunity sample of patients attending the Neurology and Neurosurgical Clinics were invited to participate in the study. All had been diagnosed with TLE on the basis of clinical presentation and instrumental diagnostic procedures, inclusive of ambulatory EEG and neuroimaging, and after failing medical therapy, had undergone surgical treatment. All patients had normal or corrected to normal visual acuity. Those with an estimated full-scale IQ of <75, as assessed with an Arabic version of the Wechsler Abbreviated Scale of Intelligence, Second Edition (WASI-II; Al-Joudi et al., 2019), were excluded, as well as those with a history of traumatic brain injury or major psychiatric disorders. Patients who suffered a seizure in the preceding 48 h had the testing session postponed. Thirteen patients took part in this study. The study’s neurosurgeon identified the anatomic structures involved by the resection on the basis of the surgical record and postsurgical MRI scans.

Fifteen healthy participants were concurrently recruited from the local community as controls. Potential participants were excluded if they had a history of a major neurologic or psychiatric disorder or an uncorrected visual impairment or an estimated IQ <75.

### Testing protocol

Testing took place in a quiet, dimly lit room. Participants sat comfortably at a distance of ∼70 cm from a MacBook Pro set at a resolution of 1680 × 1050 pixels. Custom-coded MATLAB (MathWorks) scripts used a set of freely available routines (Brainard, 1997) to control the timing of the displays. Two tasks were conducted using the computerized set-up.

### Cued color recall

**Figure 1.**
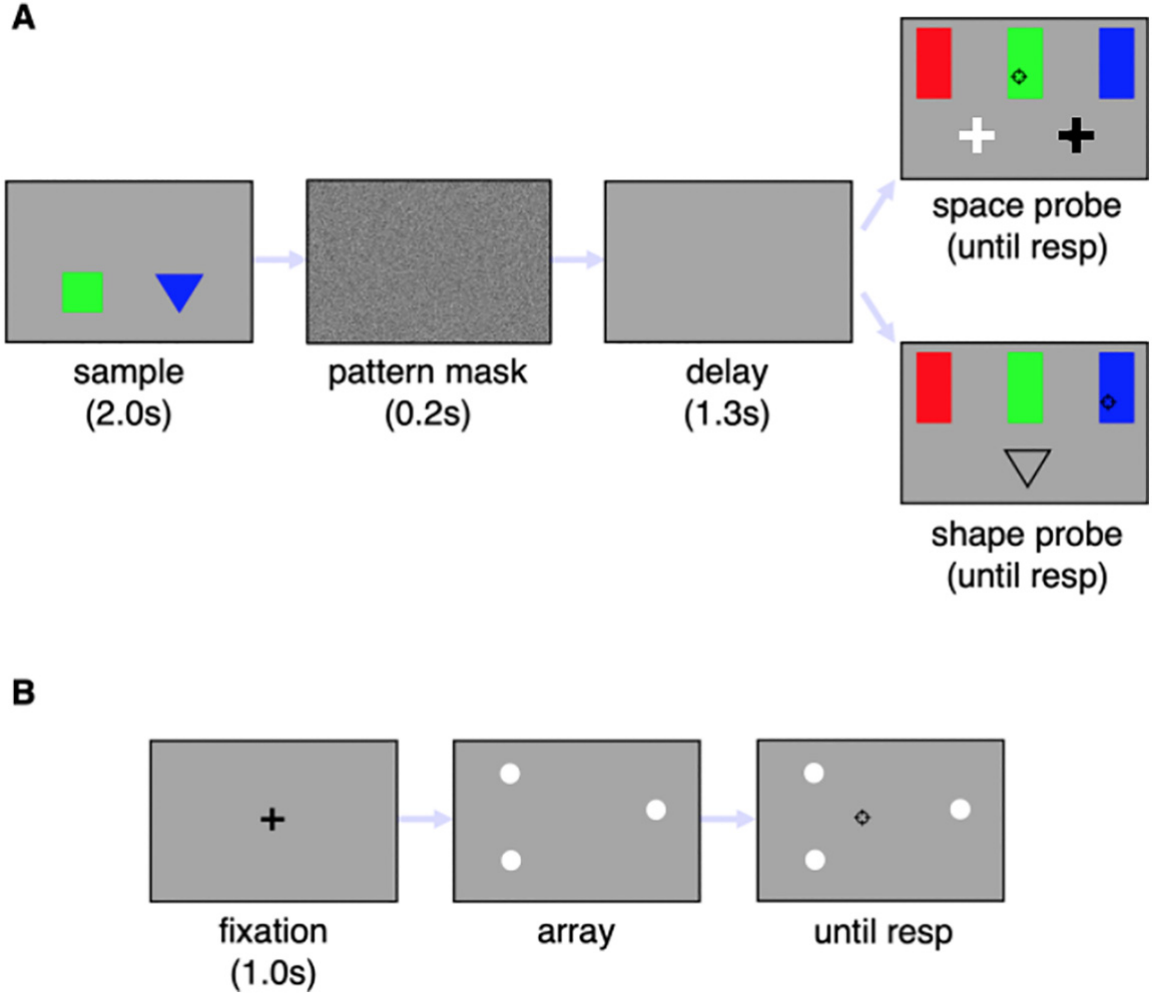
Panel ***A*** shows the event sequence in the color recall task. Participants had to remember the color of a triangle and a square displayed side by side. The sample display was followed by a pattern mask and a blank screen. The recall target was identified either by a space probe, consisting a bright cross displayed at the location previously occupied by the target, or by a shape probe, consisting of the outline of the target shape . The color was reported by placing the cursor over the corresponding rectangle and clicking the mouse button. The response (resp) initiated the next trial. Panel ***B*** shows the centroid estimation task. The visual display contained three bright dots, and the participant had to indicate the location of the center of mass of the imaginary triangle whose vertices corresponded to the dots location, by dragging the cursor and clicking.

In each trial, an equilateral triangle and a square, whose side lengths are 1.29° and 0.92°, respectively, appear side-to-side in the lower half of the screen, as shown in Figure 1*A*. The shapes are centered at an eccentricity of 2.27° along the main screen diagonals and remain visible for 2.0 s. The two shapes are of different colors, either red, blue, or green. The sample display is followed by a 0.2-s-long pattern mask and a 1.3-s blank screen. The recall screen contains three colored rectangles, 0.53° wide and 1.59° high, whose lower edges are aligned 1.39° above the screen center and spaced horizontally 4.81° apart. A bright cross (location probe) or the outline of one of the polygons (shape probe) identifies the target. The location probe, which also includes a dark cross, appear at the locations occupied by the two shapes. The shape cue appears 3.0° below the screen center. Participants report the target color by placing the cursor over the corresponding colored rectangle and clicking the mouse button. The mouse click prompts the beginning of a new trial after a 1.0-s delay during which the screen is blank. Participants practiced the task over 16 trials and then completed two blocks of 96 trials each, including both shape and location cued recalls. Trial order was randomized, minimizing participants’ ability to predict whether a shape or location probe would follow the sample display. To ensure that patients had not forgotten the task instructions, at the end of each block they were asked to describe what they had done.

### Centroid estimation

This task assesses the accuracy and precision of estimates of the average location of three visible white disks and is illustrated in Figure 1*B*. Each disk’s diameter is 0.27°. Participants place a crosshair-shaped cursor at the estimated centroid location and click the mouse. Following a 1.0-s interval, a novel set of disks appears and the procedure is repeated. Disks can occupy any three of seven canonical locations, including the screen center and the vertices of a virtual concentric hexagon, with a side length of 3.67°. All permutations of three canonical locations, less any resulting in a collinear configuration, are used as test arrays. Each possible permutation appears twice, for a total of 64 trials. Pseudorandom, zero mean, independent circular Gaussian distributions, with a SD of 0.6°, are sampled to jitter each disk’s position. Before testing, instructions were read to the participants. The centroid was defined as the point where the triangle, whose vertices coincided with the disks’ locations, would balance in the horizontal plane (Baud-Bovy and Soechting, 2001). Participants completed 25 practice trials, without feed-back, followed by two blocks of 64 trials each.

### Neuropsychological tests

Three neuropsychological instruments were used to assess participants: (1) Hopkins Verbal Learning Test–Revised (HVLT-R), (2) Brief Visuospatial Memory Test–Revised (BVMT-R), and (3) Color Trails Test (CTT). The Arabic version of these tests were recently validated (Al-Joudi et al., 2019).

### Data analysis

In the recall task, participants could either report (1) the color of the target, correct response; (2) the color of the nontarget item, that is make a swap error; or (3) the color absent from the sample, that is make a generic error. We approached the group level hypothesis testing as a metanalysis of prevalence data, treating each participant as a separate study. All inferential analysis presented in this study is Bayesian.

### Group differences in recall error rates

Group level effects were analyzed with mixed Bayesian ANOVAs using the JASP software (JASP Team, 2021; https://jasp-stats.org/). The between group variable coded whether the participant was (1) a healthy control, (2) a patient following left, or (3) right temporal lobectomy, respectively. The within group variables were probe dimension, i.e., whether a shape or space probe was used to cue recall, and block order, i.e., first or second block. The error rates were normalized with a Friedman–Tukey double arcsine transformation (Barendregt et al., 2013). The transformation stabilizes error rates variances and allows the use of parametric methods to compute group statistics:

$$t=\sin^{-1}\sqrt{\frac{c}{n}}+\sin^{-1}\sqrt{\frac{c}{n+1}},$$

where ***c*** is the number of either swap or generic errors and *n* is the total number of trials for each probe dimension and block. The group average proportions were obtained by applying the following inverse transformation to the transformed proportions group means:

$$\hat{p}=\left[1-sgn\left(\cos\hat{t}\right)\cdot \sqrt{1-{\left(\sin\hat{t}-\frac{\sin\hat{t}-1/\sin\hat{t}}{n}\right)}^{2}}\right]\cdot 0.5.$$

### Analysis of centroid task

The analysis of the centroid task was conducted by fitting the following regression model to each participants’ reports:

$$\left(\begin{array}{c} r_{x}\\ r_{y} \end{array}\right)=\left(\begin{array}{c} a_{0}\\ b_{0} \end{array}\right)+\left(\begin{array}{cc} a_{1} & 0\\ 0 & b_{1} \end{array}\right)\left(\begin{array}{c} C_{x}\\ C_{y} \end{array}\right)+\left(\begin{array}{c} N_{x}\\ N_{y} \end{array}\right),$$

where ***r****_x_* and ***r****_y_* are the *x-y* screen coordinates of the centroid estimates, ***C****_x_*_,_ ***C****_y_* are the centroid horizontal and vertical screen coordinates, and ***N****_x_* and ***N****_y_* are the normal distributions of the respective residuals. Although incenter biases are also known to affect centroid estimates (Baud-Bovy and Soechting, 2001), we did not include them in the model for sake of clarity and because a preliminary analysis did not reveal appreciable group differences. Group and screen coordinates differences in static offsets, i.e., *a_0_* and *b_0_*, accuracy, i.e., *a_1_* and *b_1_*, and precision, i.e., the variance of ***N****_x_* and ***N****_y_*, were assessed with Bayesian mixed ANOVAs.

## Results

### Participants’ demographic, clinical, and neuropsychometric characteristics

Table 1 reports the demographic and clinical characteristics of the left and right temporal lobectomy patients and healthy controls. The groups were matched on age, gender, and educational level. Both patient groups showed a lower full-scale IQ that healthy controls; however, all of the variables were more likely to reflect a null effect than a group difference. Table 2 details the sex, education level, and neuropsychometric performance of the 13 patients. Both raw scores as well the values normalized on the basis of a reference sample of healthy controls, whose first language is Arabic (Al-Joudi et al., 2019), are shown. We examined whether patients showed a material specific pattern of lateralized deficits (Saling, 2009) by comparing performance of the left and right temporal lobectomy patients on the HVMT and the BVMT with Bayesian independent samples *t* tests (JASP Team, 2021; https://jasp-stats.org/). There was moderate strength evidence for left temporal lobectomy patients having worse delayed verbal recall on the HVLT than controls (BF_10_ = 7.99), while there was anecdotal evidence for no group difference in the delayed visuo-spatial recall (BF_10_ = 0.46). In Table 3, the MTL structures affected by the surgical excisions are listed, patient by patient, while Extended Data Table 3-1 shows representative postsurgical axial, sagittal, and coronal multimodal MRI slices for each patient.

**Table 1:** Demographic and clinical sample characteristics

	left TLE (*n = *6)	right TLE (*n = *7)	controls (*n = *15)	BF_10_
Sex (%males)	83.3	100	93.3	0.64
Age (years)	35.2 (±7.9)	33.08 (±8.9)	33.3 (±7.8)	0.29
Education (highest grade)	15.3 (±1.6)	14.3 (±1.9)	14.3 (±2.4)	0.5
Full Scale IQ	92.0 (±11.7)	87.3 (±9.9)	99.5 (±15.5)	0.9
Epilepsy onset age (years)	11.1 (±10.2)	22.1 (±17.2)	—	0.84

Frequency group differences were compared using a Bayesian contingency table. Continuous variables were compared with Bayesian ANOVAs or Bayesian independent samples *t* test. The values in parenthesis are SDs. None of the demographic or clinical variables showed appreciable group differences since the Bayes factor (BF_10_) was less than 1.0 for all comparisons.

**Table 2:** Demographics and neuro-psychometric performance of TLE patients

Patient	Gender	Education (years)	WAIS II				HVLT			BVMT			CTT	
			Block design	Vocab	Matrix reasoning	Similar	Immediate	Delayed	Discrimin.	Immediate	Delayed	Discrimin.	CTT1 (s)	CTT2 (s)
P1	M	16	16 (−0.59)	18 (−1.6)	12 (−0.41)	24 (−0.77)	19 (−1.43)	6 (−1.47)	9 (−1.9)	12 (−1.01)	4 (−1.22)	4 (−1.4)	85 (1.24)	160 (2.0)
P2	M	14	18 (−0.46)	27 (−0.83)	12 (−0.41)	23 (−0.88)	26 (0.36)	9 (0.11)	12 (1.1)	10 (−1.28)	4 (−1.22)	4 (−1.4)	97 (1.74)	166 (2.22)
P3	F	16	12 (−0.88)	25 (−1.0)	18 (0.53)	28 (−0.31)	24 (−0.15)	7 (−0.95)	10 (−0.9)	9 (−1.41)	6 (−0.58)	5 (−0.4)	65 (0.41)	130 (0.92)
P4	M	14	16 (−0.59)	26 (−0.91)	14 (−0.09)	26 (−0.54)	22 (−0.67)	7 (−0.95)	11 (0.1)	14 (−0.75)	5 (−0.9)	4 (−1.4)	66 (0.45)	191 (3.12)
P5	M	12	14 (−0.74)	28 (−0.74)	10 (−0.72)	25 (−0.66)	24 (−0.15)	8 (−0.42)	8 (−2.9)	7 (−1.68)	3 (−1.55)	3 (−2.4)	65 (0.41)	135 (1.1)
P6	M	16	24 (−0.0)	20 (−1.48)	16 (0.22)	22 (−1.0)	24 (−0.15)	7 (−0.95)	11 (0.1)	21 (0.19)	7 (−0.26)	5 (−0.4)	75 (0.82)	150 (1.65)
P7	M	16	14 (−0.74)	28 (−0.74)	8 (−1.03)	25 (−0.66)	19 (−1.43)	5 (−2.0)	7 (−3.9)	12 (−1.01)	5 (−0.9)	4 (−1.4)	87 (1.33)	154 (1.79)
P8	M	16	39 (1.09)	45 (0.71)	21 (1.0)	30 (−0.08)	27 (0.62)	10 (0.63)	12 (1.1)	13 (−0.88)	9 (0.39)	5 (−0.4)	32 (−0.97)	85 (−0.7)
P9	M	14	27 (0.21)	32 (−0.4)	18 (0.53)	26 (−0.54)	21 (−0.92)	8 (−0.42)	11 (0.1)	21 (0.19)	7 (−0.26)	5 (−0.4)	52 (−0.13)	105 (0.02)
P10	M	14	19 (−0.37)	22 (−1.26)	9 (−0.88)	24 (−0.77)	18 (−1.69)	7 (−0.95)	10 (−0.9)	17 (−0.35)	7 (−0.26)	4 (−1.4)	94 (1.62)	165 (2.19)
P11	M	11	26 (0.14)	22 (−1.26)	16 (0.22)	24 (−0.77)	20 (−1.18)	7 (−0.95)	10 (−0.9)	15 (−0.61)	6 (−0.58)	4 (−1.4)	80 (1.03)	151 (1.68)
P12	M	12	13 (−0.81)	23 (−1.17)	12 (−0.41)	22 (−1.0)	19 (−1.43)	5 (−2.0)	8 (−2.9)	12 (−1.01)	5 (−0.9)	4 (−1.4)	86 (1.28)	153 (1.75)
P13	M	16	17 (−0.52)	28 (−0.74)	11 (−0.56)	28 (−0.31)	21 (−0.92)	8 (−0.42)	10 (−0.9)	14 (−0.75)	6 (−0.58)	4 (−1.4)	83 (1.16)	158 (1.94)

Raw scores are reported for each test and participant (see Materials and Methods). The corresponding normalized values are shown in parenthesis. Normalized *z* scores values were computed by subtracting the mean score and dividing by the SD of a reference sample (Al-Joudi et al., 2019). HVLT-R = Hopkins Verbal Learning Test–Revised; BVMT-R = Brief Visuospatial Memory Test–Revised; CTT = Color Trails Test.

**Table 3:** Patients’ lesion anatomy

	Age	Pathology	Lesion laterality	Temporal lobe structures								
				HIP	ERC	PRC	PHC	ITG	MTG	TP	STG	AMG
P1	25	GGs	L	0	+	+	0	0	0	+	0	0
P2	25	MTS	R	++	0	0	0	0	0	+	0	++
P3	50	MG	L	0	0	+	0	0	+	+	0	0
P4	41	GGs	R	0	+	+	0	0	0	+	0	0
P5	31	MTS	R	++	+	0	+	0	+	+	+	++
P6	27	MTS	L	++	0	0	0	0	0	0	0	0
P7	40	MG	L	0	0	0	0	0	+	+	0	0
P8	32	MTS	R	++	0	0	0	+	0	++	0	+
P9	33	MTS	R	++	+	0	++	+	0	0	+	+
P10	22	MTS	L	+	+	0	0	++	0	0	+	0
P11	25	MTS	R	++	+	0	0	0	0	0	+	++
P12	47	GGs	L	0	+	0	0	0	++	++	+	0
P13	32	MTS	R	+	+	0	+	+	0	0	+	0

The table lists the pathology and regions affected by the lobectomy for each patient. Extended Data Table 3-1 shows representative MRI slices through the MTL. GG, ganglioglioma; MG, meningioma; MTS, medial temporal sclerosis; HIP, hippocampus; ERC, entorhinal cortex; PRC, perirhinal cortex; PHC, parahippocampal cortex; ITC, inferotemporal cortex; MTG, Middle temporal gyrus; ATP, anterior temporal pole; STG, superior temporal gyrus; AMG, amygdala. 0 indicates an unaffected subregion, + a rostro-caudal lesion extent up to 20 mm, and ++ up to 40 mm.

10.1523/ENEURO.0278-21.2022.tab3-1 Extended Data Table 3-1 Postsurgical MRI scans for 13 patients. The images were obtained with T1 weighted, T2 weighted, fluid-attenuated inversion recovery, and gradient recalled echo sequences. ERC, entorhinal cortex; PHC, parahippocampal cortex; PRC, perirhinal cortex; Hipp, hippocampus; AMG, amygdala; TP, temporal pole; antSTG, anterior superior temporal gyrus; antMTG, anterior middle temporal gyrus. Download Table 3-1, DOCX file.

### Cued color recall performance

In the cued recall task, participants completed two blocks of 96 trials each. Mixed effect Bayesian ANOVAs were used to examine the influence of three factors on recall: (1) group, namely, whether participants were controls, patients following left and right temporal lobectomy, respectively; (2) block; and (3) probe dimension. Generic and swap errors were analyzed separately.

Extended Data Table 4-1 reports the result of the ANOVA model comparison for swap errors. The model with the highest posterior probability included group, probe dimension, and block, as well as the interaction of group by probe dimension. Table 4 summarizes the evidence for each predictor. There was moderate evidence for an effect of probe dimension and block. There was very strong evidence for an effect of group and strong evidence for an interaction of group by probe dimension.

**Table 4:** Swap errors, analysis of effects

Effects	P(incl)	P(incl|d)	BF_incl_
B	0.737	0.941	**5.673**
D	0.737	0.925	**4.414**
G	0.737	0.997	**119.755**
B·D	0.316	0.206	0.563
G·B	0.316	0.188	0.500
G·D	0.316	0.891	**17.629**
G·B·D	0.053	0.017	0.317

The table lists each factor and interaction for swap error rates. Extended Data Table 4-1 lists the models and associated prior and posterior probabilities from which the values in the present table are computed. P(incl) is the prior probability of the effect; P(incl|d) is the posterior probability of the effect; BF_incl_ is Bayes factor. A BF greater than 1.0 favors the effect, a BF less than 1.0 favors a null instead. Values of the BF greater than 3.0 are in bold, to highlight those effects that have at least moderate evidence in their favor. Block, probe dimension, group, and the interaction of group by probe dimension all have at least moderately strong evidence in their favor.

10.1523/ENEURO.0278-21.2022.tab4-1 Extended Data Table 4-1 Best model comparisons for swap errors. The table presents each of the model comparisons from the Bayesian ANOVA. The within factors are block (B) and probe dimension (D). The between factor is group G. P(M) is the a priori model probability, P(M|d) is the posterior model probability. BF_M_ is the Bayes factor of the model, BF_10_ is the Bayes factor of the model relative to the best one. The best model contained all three factors and the interaction of group by probe dimension. Download Table 4-1, DOC file.

Extended Data Table 5-1 summarizes the results of the model comparisons for generic errors. The best model was the one which included all three factors, but none of the interactions. Table 5 summarizes the analysis of the effects. There was anecdotal evidence for the block and probe dimension affecting the proportion of generic errors. On the other hand, there was anecdotal evidence for a null effect of group.

**Table 5:** Generic error, analysis of effects

Effects	P(incl)	P(incl|d)	BF_incl_
B	0.737	0.804	1.463
D	0.737	0.815	1.571
G	0.737	0.641	0.637
B·D	0.316	0.142	0.360
G·B	0.316	0.085	0.202
G·D	0.316	0.087	0.206
G·B·D	0.053	7.157e −4	0.013

The table lists each factor and interaction for generic error rates. Extended Data Table 5-1 lists the models and associated probabilities, used to compute the effects. P(incl) is the prior probability of the predictors; P(incl|d) is the posterior probability; BF_incl_ is Bayes factor. A BF greater than 1.0 favors the predictor, a BF less than 1.0 favors a null effect instead. The only predictors with favorable evidence, albeit of very modest entity, are block and probe dimension.

10.1523/ENEURO.0278-21.2022.tab5-1 Extended Data Table 5-1 Best model comparisons for generic errors. The table presents the model comparisons obtained from a Bayesian ANOVA. The within factors are block (B) and probe dimension (D). The between factor is group G. P(M) is the a priori model probability, P(M|d) is the posterior model probability. BF_M_ is the Bayes factor of the model, BF_10_ is the Bayes factor of the model relative to the best one. The best model included the three main factors, namely, block (B), probe dimension (D), and group (G). Download Table 5-1, DOC file.

**Figure 2.**
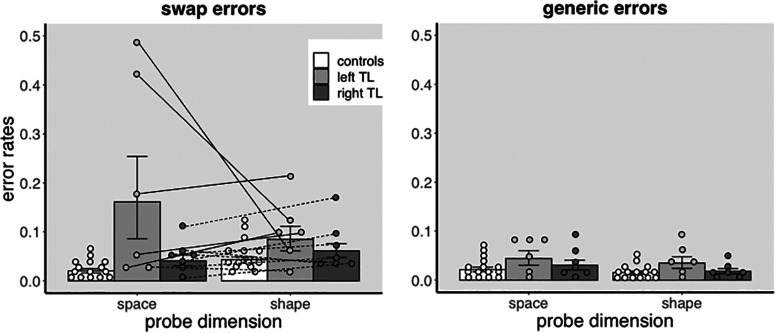
Recall error rates. The bar graph on the left shows the group averaged proportions of swap errors, while the bar graph on the right shows the group averaged proportions of generic errors, following space and shape probes, respectively. Overall, patients made more swap errors than controls. Moreover, patients with left temporal lobectomies made more swap errors following space than shape probes, suggesting a specific impairment of spatial binding in this group only. For generic errors, group differences were marginal and were not further affected by probe dimension. For sake of clarity, the data are averaged over the two blocks. Circles are individual participants’ error rates. Continuous lines join swap error rates, following space and shape probes, respectively, of each left temporal lobectomy patient. Broken lines join the swap error rates of each right temporal lobectomy patient. Error bars are SEM.

Figure 2 shows the mean proportion of swap and generic errors following space and shape probe, respectively. Controls made fewest swap and generic errors. Controls and patients with right temporal lobectomies made more swap errors following shape than space probes. Patients with left temporal lobectomies made most swap errors and three of them made more swap errors following space than shape probes. Participants made more generic errors following space than shape probe. Participants also made more swap and generic errors in the first than second block (data not shown).

### Centroid estimation performance

We examined how group and screen coordinates affected three indices of performance in the centroid estimation task: (1) static offsets, (2) accuracy, and (3) precision (see Materials and Methods). For static offsets the model with the highest posterior probability included the effect of screen coordinates only. In fact, there was strong evidence (BF_inc_ = 32.13) that the horizontal and vertical offsets differed. While there was no appreciable horizontal bias, *m *=* *0.0°, 95%CI = [−0.04°, 0.05°], participants showed an upward bias, *m *=* *0.15°, 95%CI = [0.07°, 0.23°]. There was moderate evidence in favor of the null and against both an effect of group (BF_inc_ = 0.22), and its interaction with coordinate (BF_inc_ = 0.28). For accuracy, the model with highest posterior probability included group. However, there was anecdotal evidence for a null effect of group (BF_inc_ = 0.76), with moderate evidence in favor of a null effect of coordinate (BF_inc_ = 0.26) and its interaction with group (BF_inc_ = 0.28). For precision, namely, the variance of the variable errors, the null model had the highest posterior probability. There was anecdotal evidence for a null effect of screen coordinate (BF_inc_ = 0.4), anecdotal evidence for a null effect of group (BF_inc_ = 0.53), and moderate evidence for a null interaction of group and dimension (BF_inc_ = 0.15).

## Discussion

In this study, we compared performance of healthy controls and temporal lobectomy patients in two tasks, one probing conjunctive binding in vWM, the other perceptual, spatial averaging of disks patterns. The vWM task required the recall of a target’s color, where the target was identified by either a location or a shape probe. The task was thus designed to determine dimensional specificity of WM conjunctive binding. Controls were more accurate than patients overall. Moreover, they made fewer swap errors following space than shape probes, while left temporal lobectomy patients made more swap errors following space than shape probes. There was no evidence of group differences in static biases, accuracy and precision of perceptual centroid estimates. The implication of these findings for the organization of binding and spatial processes in vWM is discussed in the next paragraph, following a brief overview of prior evidence.

### MTL lesions specifically disrupt spatial binding in vWM

Previous studies addressed whether MTL pathology is associated with impairments in vWM binding. The ability to recall shape-color, shape-location or item-item conjunctions has been found to be diminished in patients with anoxic/ischemic or infectious pathology involving the MTL as well as neurodegenerative disorders, suggesting an impairment in conjunctive and relational binding (Hannula et al., 2006; Olson et al., 2006; Parra et al., 2009). van Geldorp et al. (2014) compared patients, who had undergone anterior temporal lobectomies for medically refractory epilepsy, and healthy controls’ performance in four match-to-sample tasks. The tasks were difficult and required participants to remember three separate frames presented sequentially. Each frame contained the picture of a face and a building, which differed in location and color. A cue, presented before the sample, indicated whether participants should only remember the identity of the items or also their location (spatial binding condition), color (color binding condition), or the item they had been presented with (relational binding condition). Overall, recall was less accurate in patients compared with controls, and particularly so in the relational binding condition. Recall performance in the spatial and color binding conditions were equally affected, suggesting that spatial and nonspatial WM binding were not differentially compromised in these patients. Zokaei et al. (2019) found that patients who had undergone a temporal lobectomy made more swap errors when recalling the location of fractal patterns, compared with controls. Since neither fractal recognition nor memory for locations were found to be appreciably impaired in these patients, it was inferred that they suffered a primary binding deficit. Using a similar paradigm, Pertzov et al. (2013) documented both a spatial as well as a nonspatial binding deficit in individuals recovering from autoimmune encephalitis, suggesting that binding impairments because of MTL dysfunction are not dimensionally specific. Braun et al. (2011) concluded instead that TLE patients, who had undergone a right temporal lobectomy, were only impaired when the vWM task required spatial binding but performed similarly to healthy controls when it required binding of nonspatial features.

Our own findings contribute new, crucial evidence for understanding the role of MTL in vWM binding by confirming the association between MTL pathology and spatial binding impairments, unaccounted for by impairments of either spatial vision or feature memory. We found an increase in the proportion of swap errors in the TLE group. Crucially, while healthy participants made significantly more swap errors following the shape probe, left temporal lobectomy patients made more swap errors following the space probe. In healthy participants the results are therefore in keeping with the hypothesis that binding of nonspatial features is mediated by the features’ shared location (Treisman and Zhang, 2006; Schneegans and Bays, 2017). In fact, the likelihood of swap errors should be greater following shape compared with space probes, because in the latter case both target shape and color need to be bound to the target location before they can be bound to each other (Schneegans and Bays, 2017). On the other hand, the observation that some patients made significantly more swap errors following space probes than controls may indicate that patients gained the ability to bind nonspatial features directly, without the mediation of a shared location, allowing them to achieve higher accuracies following shape than space probes. Whether this inference is warranted remains to be established. Regardless, the group level pattern of dimensionally specific binding impairments observed in left TLE patients replicates a previous observation in a stroke patient with bilateral MTL damage, found to be impaired only in vWM tasks requiring spatial binding, but not those requiring nonspatial binding (Dundon et al., 2018). These observations thus allow us to draw the following conclusion: MTL pathology can be associated with WM binding impairments that are spatially specific and reverse the spatial advantage characteristic of healthy controls. If processes underlying spatial binding in vWM are independent from processes devoted to binding of nonspatial features, then the role of space in organizing vWM may extend beyond providing a common index for the conjunctive binding of visual features.

### Hemispheric lateralization and binding

Bohbot et al. (1998) found that patients who had undergone thermocoagulation of structures within the right, but not the left, MTL were more impaired in a number of spatial WM tasks than those who had not undergone surgery, suggesting that right MTL structures may play an overarching role in spatial memory. Braun et al. (2011) compared patients with right temporal lobectomy and healthy controls’ performance on a number of single feature and feature conjunction recall tasks and concluded that these patients are specifically impaired in spatial binding. However, the tasks employed memory samples of different complexity to probe recall of features and conjunctions, respectively, thus introducing a load confound in the comparison. Our own results are in keeping with the idea that left rather than right MTL structures are specifically involved in spatial binding, since patients who had undergone left temporal lobectomies showed greater spatial binding impairments than patients with right temporal lobectomies. While our results need to be interpreted cautiously, given the small sample size, they agree with the conclusion drawn by Kessels et al. (2004), who found that patients who had undergone left, but not right temporal lobectomies were impaired in spatial binding, confirming lateralization effects previously observed by the same group in a sample of patients with cerebrovascular pathology (Kessels et al., 2002). Spiers et al. (2001) found that left temporal lesion specifically affect object location binding while right temporal lesions affect spatial memory more generally. However, the existing literature remains inconclusive to the relation between laterality and WM spatial binding. A distinct view of lateralization of binding impairments is that the latter reflect attention deficits which follow cortical strokes, especially involving the nondominant right hemisphere. For example. Cohen-Dallal et al. (2021) reported that patients with unilateral attentional neglect show delay-dependent decrements in spatial binding performance. In light of the fact that parahippocampal damage is associated with unilateral neglect (Mort et al., 2003), Cohen-Dallal et al. (2021)’s finding raises the possibility that lateralized attentional deficits may also contribute to the binding impairments observed in our study. However, performance in a centroid estimation task did not show group differences in lateralized static biases, suggesting that lesion in our sample was not associated with unilateral neglect. Moreover, patients showed neither diminished accuracy nor lower precision in the centroid task compared with controls, indicating that spatial perception and attention were not compromised (Drew et al., 2010).

A further concern is that uncontrolled verbal strategies may have confounded the interaction of lesion laterality and probe dimension. We found in pilot studies that healthy participants maintain a spatial advantage in WM binding under condition of articulatory suppression and therefore concluded that the spatial advantage in WM binding does not depend on verbal strategies (d’Avossa G, Dundon NM, Katshu MZUH, unpublished data). However, we cannot rule out the possibility that left temporal lobectomy patients used a verbal strategy and thus managed to selectively improve binding of shape and color.

It is important to note, with regard to the issue of localization and the nature of cognitive impairments encountered in TLE patients, that group level results belie substantial interindividual differences (see Fig. 2). Previous electrophysiological recordings from the left MTL indicated that trial by trial changes in the amplitude of low-frequency oscillations, obtained during encoding episodes, predict subsequent recall accuracy of object/place conjunctions in TLE patients (Miller et al., 2018), in keeping with our own conclusion that spatial binding is dependent on left lateralized processes. Interestingly, this was not the case in all suggesting that spatial binding processes are not universally left lateralized in these patients. Unfortunately, the study did not report whether the lateralization of spatial binding processes was affected by the laterality of the seizure focus, precluding firmer conclusion regarding the relation between the two. Other studies have, however, demonstrated anomalous lateralization in high proportion of TLE patients, as memory processes often shift to the contralesional hemisphere both preoperatively (Bellgowan et al., 1998; Golby et al., 2002; Janszky et al., 2004) and postoperatively (Sidhu et al., 2016). These findings may provide one possible interpretation of the interindividual differences highlighted above, namely, that neural plasticity in some TLE patients modifies the lateralization of memory processes usually encountered in healthy controls. An alternative explanation, initially born out of observations in nonhuman primates with focal lesions (Browning et al., 2010; Croxson et al., 2012), is that effects of MTL functional and structural damage in postsurgical TLE patients may be attenuated by nonlateralized recruitment of neocortical areas (Sidhu et al., 2013). To determine whether either or both of these hypotheses can account for interindividual differences in spatial binding impairments and to what extent other factors, like age of seizure onset, severity and frequency of seizures, neuropsychiatric complications, and antiepileptic medications, known to affect neural plasticity and degree of cognitive impairment (Bell et al., 2011), also contribute to hemispheric lateralization in TLE patients will require new experimental evidence.

Despite the potential confound listed above, the present study indicates that following left temporal lobectomy, vWM binding is diminished in a dimensionally specific manner in the absence of appreciable perceptual, attentional, or visual-spatial memory deficits.

## Synthesis

Reviewing Editor: Alexander Soutschek, Ludwig-Maximilians-Universitat Munchen Decisions are customarily a result of the Reviewing Editor and the peer reviewers coming together and discussing their recommendations until a consensus is reached. When revisions are invited, a fact-based synthesis statement explaining their decision and outlining what is needed to prepare a revision will be listed below. The following reviewer(s) agreed to reveal their identity: Sebastian Schneegans, Paul Bays.

The revised manuscript replaces the previous statistical analysis with a Bayesian ANOVA, which is both easier to follow and more appropriate for the presented data. This addresses the main criticism for the original version of the manuscript.

One suggestion for improvement that the reviewers still have is that the variability in outcome for the left temporal lobectomy patients should be addressed more explicitly. Three of the patients show performance in the space cue task that is within the range of healthy controls, while three others show substantial impairments. The reviewers don’t think that this variability constitutes any kind of weakness of the results, but it is a salient aspect and important for the interpretation of the findings, and it deserves to be described and discussed. The authors did include a brief discussion of inter-individual differences at the end of the manuscript, but without addressing the specific pattern of results. It would also be interesting to report what the other performance measures for these particular individuals were (i.e., what other data points in Figure 2 belong to them).

Minor comments:

1. In the cued recall method, it states that “A bright cross” is used as location probe, but the figure shows a white dot - which is it?

2. The reason for using double arcsin transformation should be explained.

3. In the centroid task results, the CI doesn’t seem to include the mean for one outcome (m = 0.14, 95%CI = [-0.19 -- 0.1]). Also, it would clearer (and following standard mathematical notation) to give CI ranges in the form [lower, upper], i.e. with a comma instead of a hyphen.

4. The following sentence no longer applies: “We approached the group level hypothesis testing as a metanalysis [sic] of prevalence data, treating each participant as a separate study.” (line 173)

## Author Response

Dear Dr Soutschek,

on behalf of the authors, I am submitting a 2nd revision of the manuscript eN-NWR-0278-21R1 titled “Spatial Binding Impairments in Visual Working Memory following Temporal Lobectomy”.

While the reviewers were satisfied with the quality of the first revision, they had few additional suggestions to further improve the final manuscript. We have taken their suggestion in stride and have updated the current version of the manuscript accordingly. We detail below the specific changes made.

I hope you will find the change satisfactory and take the opportunity to thank you again for assisting us in the process leading to publication of our study in eNeuro.

Best Regards

Giovanni d’Avossa, MD

Reviewers’ comments and replies

One suggestion for improvement that the reviewers still have is that the variability in outcome for the left temporal lobectomy patients should be addressed more explicitly. Three of the patients show performance in the space cue task that is within the range of healthy controls, while three others show substantial impairments. The reviewers don’t think that this variability constitutes any kind of weakness of the results, but it is a salient aspect and important for the interpretation of the findings, and it deserves to be described and discussed. The authors did include a brief discussion of inter-individual differences at the end of the manuscript, but without addressing the specific pattern of results. It would also be interesting to report what the other performance measures for these particular individuals were (i.e., what other data points in Figure 2 belong to them).

Figure 2 now includes lines joining same patient swap errors rates, following space and shape probes respectively. This allows the reader to establish, in each individual patient, relative swap errors rates by visual inspection. The data of individual controls participants were not joined to avoid crowding the figure. The data concerning generic errors were also not further highlighted since generic errors were much rarer than swap errors and, more importantly, did not demonstrate any appreciable group difference or group by probe dimension interaction. The results and figure legend have been reworded to describe the updated figure 2.

We have also rewritten the part of the discussion dealing with interindividual differences and included new references. In particular, Miller at al. (2108) electrophysiological study of presurgical patients is now included. This study provided prima facie evidence that MTL processes associated with encoding spatial bindings in visual memory are left hemisphere lateralized in many, but not all TLE patients. We put forth two specific hypotheses that may account for the observed interindividual differences, based on the idea that within or between hemispheres neural plasticity produces heterogeneity in lateralization of spatial binding processes.

Minor comments:

1. In the cued recall method, it states that “A bright cross” is used as location probe, but the figure shows a white dot - which is it?

The reviewer is correct. The location cue was a cross not a cue. Figure 1 has been corrected.

2. The reason for using double arcsin transformation should be explained.

The double arcsin transformation stabilizes the variance of error rates estimates and thus allow use of parametric statistics such as the Bayesian ANOVAs. A brief sentence to this effect has been added to the methods section.

3. In the centroid task results, the CI doesn’t seem to include the mean for one outcome (m = 0.14, 95%CI = [-0.19 -- 0.1]). Also, it would clearer (and following standard mathematical notation) to give CI ranges in the form [lower, upper], i.e. with a comma instead of a hyphen.

We corrected the mistaken CI interval. We have also changed two CIs using the suggested statistical notation.

4. The following sentence no longer applies: “We approached the group level hypothesis testing as a metanalysis [sic] of prevalence data, treating each participant as a separate study.” (line 173)

We removed the sentence.
