# Supplementary material for: Spatial Binding Impairments in Visual Working Memory following Temporal Lobectomy
Source: eNeuro. 2022 Mar 8;9(2):ENEURO.0278-21.2022. doi: 10.1523/ENEURO.0278-21.2022 (PMC8906795; doi:10.1523/ENEURO.0278-21.2022)
Supplement: Extended Data Table 3-1 — Postsurgical MRI scans for 13 patients. The images were obtained with T1 weighted, T2 weighted, fluid-attenuated inversion recovery, and gradient recalled echo sequences. ERC, entorhinal cortex; PHC, parahippocampal cortex; PRC, perirhinal cortex; Hipp, hippocampus; AMG, amygdala; TP, temporal pole; antSTG, anterior superior temporal gyrus; antMTG, anterior middle temporal gyrus. Download Table 3-1, DOCX file. [file enu-eN-NWR-0278-21-s02.docx]

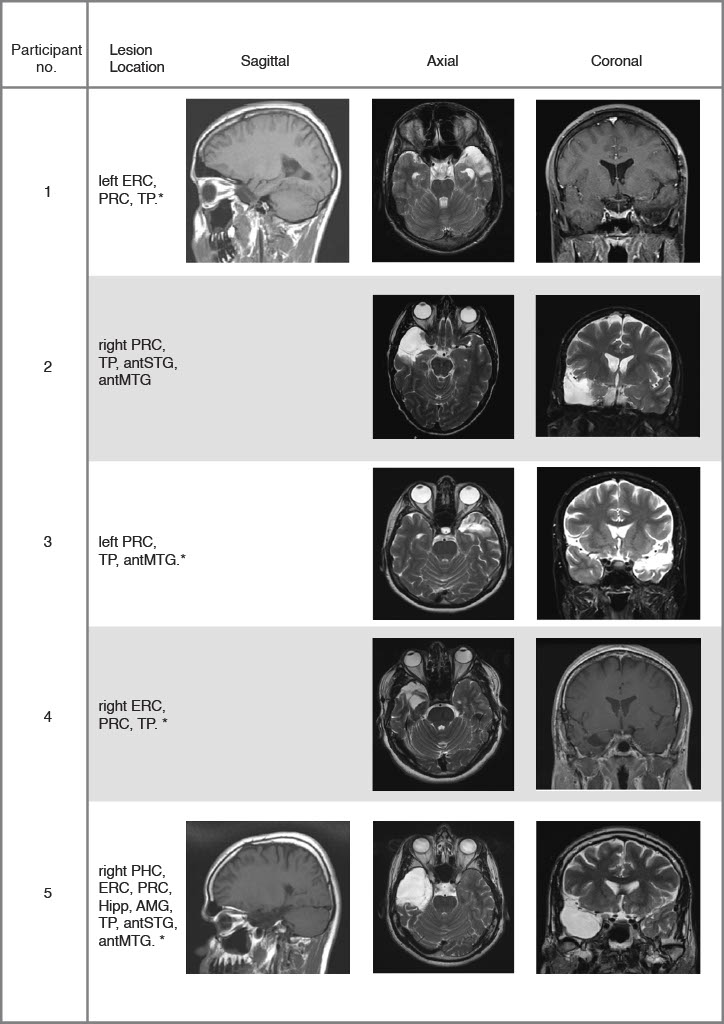


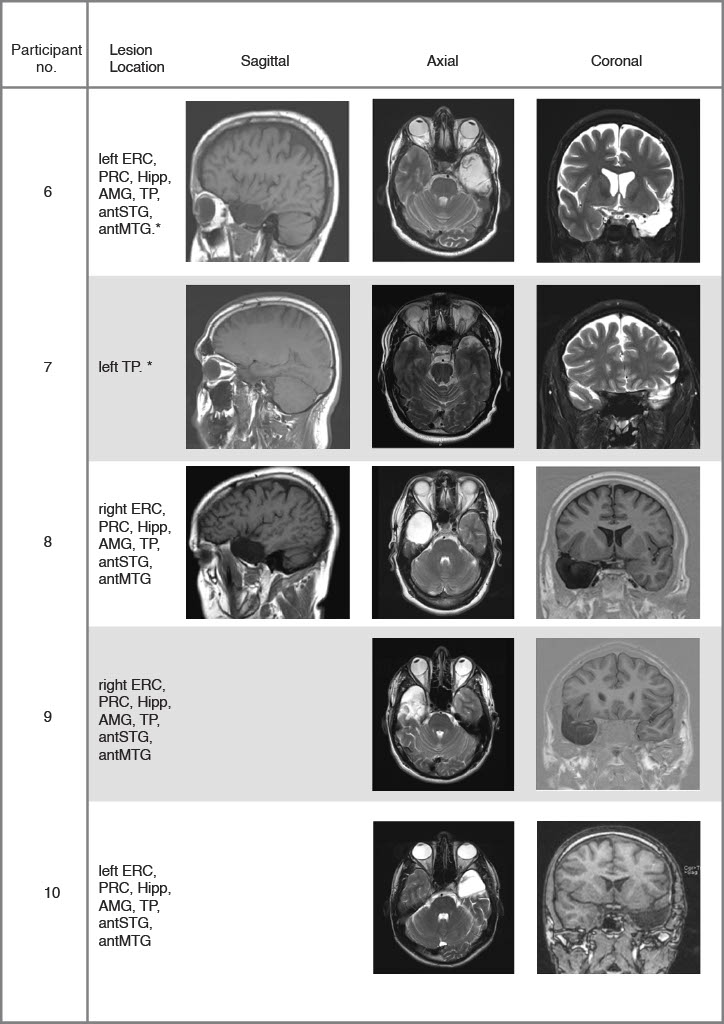


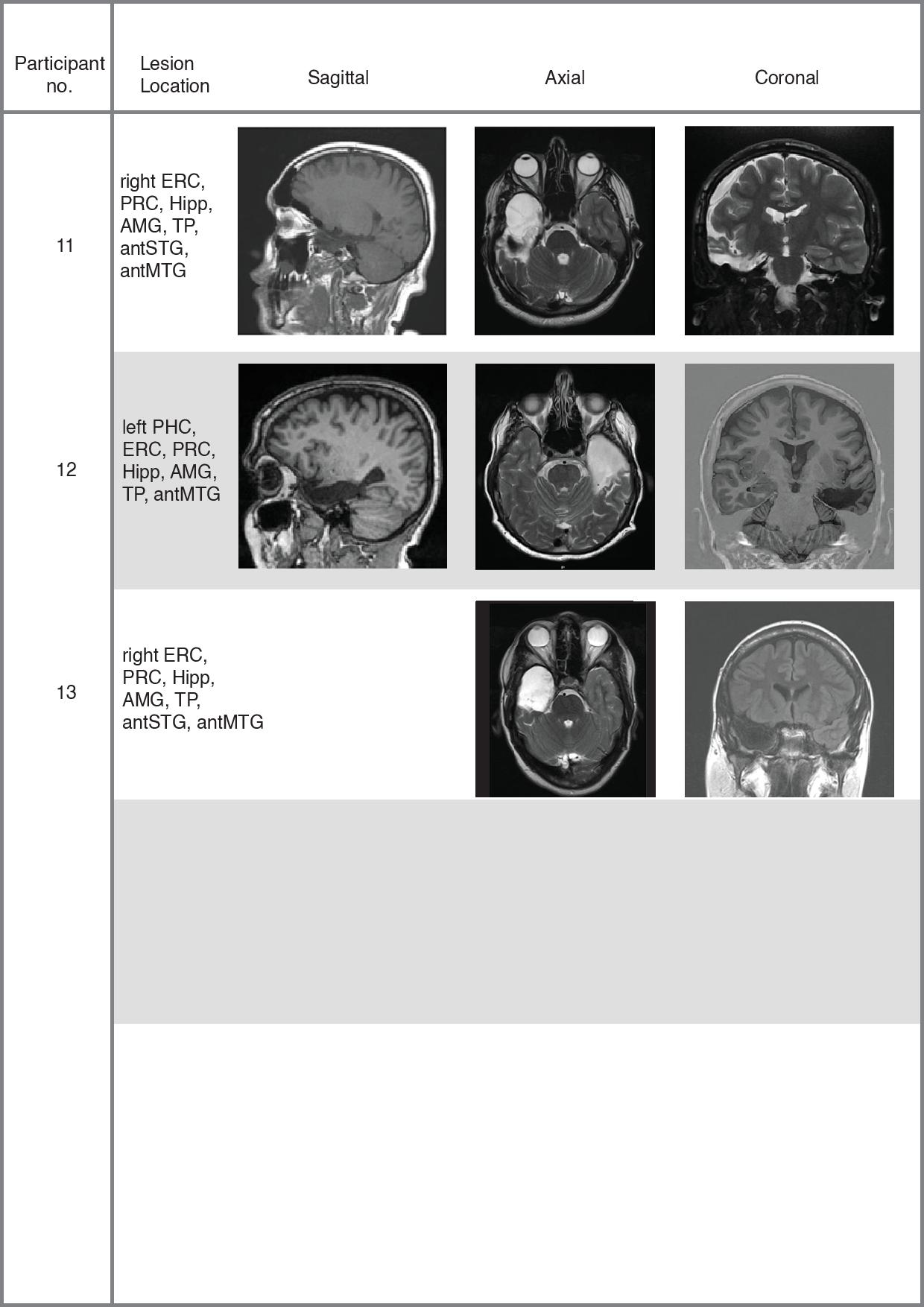


*Table 3-1 (Extended data). Post-surgical MRI scans for thirteen patients. The images were obtained with T1 weighted, T2 weighted, Fluid-attenuated Inversion Recovery and Gradient Recalled Echo sequences. Legend: ERC, Entorhinal Cortex; PHC, Parahippocampal Cortex; PRC, Perirhinal Cortex; Hipp, Hippocampus; AMG, Amygdala; Temporal pole, TP; antSTG, anterior Superior Temporal Gyrus; antMTG, anterior Middle Temporal Gyrus.*
