# Supplementary material for: Spatial Binding Impairments in Visual Working Memory following Temporal Lobectomy
Source: eNeuro. 2022 Mar 8;9(2):ENEURO.0278-21.2022. doi: 10.1523/ENEURO.0278-21.2022 (PMC8906795; doi:10.1523/ENEURO.0278-21.2022)
Supplement: Extended Data Table 4-1 — Best model comparisons for swap errors. The table presents each of the model comparisons from the Bayesian ANOVA. The within factors are block (B) and probe dimension (D). The between factor is group G. P(M) is the a priori model probability, P(M|d) is the posterior model probability. BFM is the Bayes factor of the model, BF10 is the Bayes factor of the model relative to the best one. The best model contained all three factors and the interaction of group by probe dimension. Download Table 4-1, DOC file. [file enu-eN-NWR-0278-21-s03.doc]

| **Table 4-1. Swap errors.** | | | | | | | | | | | | | | | |
| --- | --- | --- | --- | --- | --- | --- | --- | --- | --- | --- | --- | --- | --- | --- | --- |
| **Models** | **P(M)** | | | | **P(M|d)** | | | **BF_M_** | | | **BF_10_** | |  | | |
| B + D + G + D•G |  | 0.053 |  | 0.522 | |  | 19.649 | |  | 1.000 | |  | |  |  |
| B + D + G + B•D + D•G |  | 0.053 |  | 0.150 | |  | 3.175 | |  | 0.287 | |  | |  |  |
| B + D + G + B•G + D•G |  | 0.053 |  | 0.121 | |  | 2.480 | |  | 0.232 | |  | |  |  |
| B + G |  | 0.053 |  | 0.053 | |  | 1.007 | |  | 0.102 | |  | |  |  |
| D + G + D•G |  | 0.053 |  | 0.048 | |  | 0.905 | |  | 0.092 | |  | |  |  |
| B + D + G + B•D + B•G + D•G |  | 0.053 |  | 0.032 | |  | 0.604 | |  | 0.062 | |  | |  |  |
| B + D + G |  | 0.053 |  | 0.021 | |  | 0.379 | |  | 0.039 | |  | |  |  |
| B + D + G + B•D + B•G + D•G + B•D•G |  | 0.053 |  | 0.017 | |  | 0.317 | |  | 0.033 | |  | |  |  |
| B + G + B•G |  | 0.053 |  | 0.012 | |  | 0.210 | |  | 0.022 | |  | |  |  |
| G |  | 0.053 |  | 0.008 | |  | 0.149 | |  | 0.016 | |  | |  |  |
| B + D + G + B•D |  | 0.053 |  | 0.005 | |  | 0.093 | |  | 0.010 | |  | |  |  |
| B + D + G + B •G |  | 0.053 |  | 0.004 | |  | 0.073 | |  | 0.008 | |  | |  |  |
| D + G |  | 0.053 |  | 0.003 | |  | 0.050 | |  | 0.005 | |  | |  |  |
| B |  | 0.053 |  | 0.002 | |  | 0.033 | |  | 0.003 | |  | |  |  |
| B + D + G + B•D + B•G |  | 0.053 |  | 0.001 | |  | 0.021 | |  | 0.002 | |  | |  |  |
| B + D |  | 0.053 |  | 6.102e-4 | |  | 0.011 | |  | 0.001 | |  | |  |  |
| Null model (incl. subject) |  | 0.053 |  | 2.921e-4 | |  | 0.005 | |  | 5.596e-4 | |  | |  |  |
| B + D + B•D |  | 0.053 |  | 1.612e-4 | |  | 0.003 | |  | 3.089e-4 | |  | |  |  |
| D |  | 0.053 |  | 9.768e-5 | |  | 0.002 | |  | 1.872e-4 | |  | |  |  |
|  | | | | | | | | | | | | | | | |
| *Table 4-1 (Extended data). Best model comparisons for swap errors. The table presents for each model the results of the Bayesian ANOVA. The within factors are block (B) and probe dimension (D). The between factor is group G. P(M) is the a-priori model probability, P(M|d) is the posterior model probability. BF_M_ is the Bayes factor of the model, BF_10_ is the Bayes factor of the model relative to the best one. The best model contained all three factors and the interaction of group by probe dimension.* | | | | | | | | | | | | | | | |
