# Supplementary material for: Spatial Binding Impairments in Visual Working Memory following Temporal Lobectomy
Source: eNeuro. 2022 Mar 8;9(2):ENEURO.0278-21.2022. doi: 10.1523/ENEURO.0278-21.2022 (PMC8906795; doi:10.1523/ENEURO.0278-21.2022)
Supplement: Extended Data Table 5-1 — Best model comparisons for generic errors. The table presents the model comparisons obtained from a Bayesian ANOVA. The within factors are block (B) and probe dimension (D). The between factor is group G. P(M) is the a priori model probability, P(M|d) is the posterior model probability. BFM is the Bayes factor of the model, BF10 is the Bayes factor of the model relative to the best one. The best model included the three main factors, namely, block (B), probe dimension (D), and group (G). Download Table 5-1, DOC file. [file enu-eN-NWR-0278-21-s04.doc]

| **Table 5-1. Generic errors - model comparison** | | | | | | | | | | | | | | |
| --- | --- | --- | --- | --- | --- | --- | --- | --- | --- | --- | --- | --- | --- | --- |
| **Models** | | **P(M)** | | **P(M|d)** | | | **BF_M_** | | | **BF_10_** | |  | | |
| B + D + G |  | 0.053 |  | | 0.231 |  | | 5.413 |  | 1.000 |  | |  |  |
| B + D |  | 0.053 |  | | 0.204 |  | | 4.616 |  | 0.883 |  | |  |  |
| D + G |  | 0.053 |  | | 0.077 |  | | 1.505 |  | 0.334 |  | |  |  |
| B + G |  | 0.053 |  | | 0.070 |  | | 1.360 |  | 0.304 |  | |  |  |
| B + D + G + B•D |  | 0.053 |  | | 0.066 |  | | 1.264 |  | 0.284 |  | |  |  |
| D |  | 0.053 |  | | 0.053 |  | | 1.017 |  | 0.231 |  | |  |  |
| B |  | 0.053 |  | | 0.050 |  | | 0.953 |  | 0.217 |  | |  |  |
| B + D + G + B•G |  | 0.053 |  | | 0.045 |  | | 0.842 |  | 0.193 |  | |  |  |
| B + D + G + D•G |  | 0.053 |  | | 0.045 |  | | 0.840 |  | 0.193 |  | |  |  |
| B + D + B•D |  | 0.053 |  | | 0.044 |  | | 0.830 |  | 0.191 |  | |  |  |
| G |  | 0.053 |  | | 0.028 |  | | 0.524 |  | 0.122 |  | |  |  |
| Null model (incl. subject) |  | 0.053 |  | | 0.020 |  | | 0.363 |  | 0.086 |  | |  |  |
| B + D + G + B•D + B•G |  | 0.053 |  | | 0.015 |  | | 0.266 |  | 0.063 |  | |  |  |
| D + G + D•G |  | 0.053 |  | | 0.014 |  | | 0.259 |  | 0.061 |  | |  |  |
| B + G + B•G |  | 0.053 |  | | 0.014 |  | | 0.253 |  | 0.060 |  | |  |  |
| B + D + G + B•D + D•G |  | 0.053 |  | | 0.012 |  | | 0.213 |  | 0.051 |  | |  |  |
| B + D + G + B•G + D•G |  | 0.053 |  | | 0.009 |  | | 0.163 |  | 0.039 |  | |  |  |
| B + D + G + B•D + B•G + D•G |  | 0.053 |  | | 0.003 |  | | 0.046 |  | 0.011 |  | |  |  |
| B + D + G + B•D + B•G + D•G + B•D•G |  | 0.053 |  | | 7.324e -4 |  | | 0.013 |  | 0.003 |  | |  |  |
|  | | | | | | | | | | | | | | |



*Table 5-1 (Extended data). Best model comparisons for generic errors. The table presents the results of the Bayesian ANOVA. The within factors are block (B) and probe dimension (D). The between factor is group G. P(M) is the a-priori model probability, P(M|d) is the posterior model probability. BF_M_ is the Bayes factor of the model, BF_10_ is the Bayes factor of the model relative to the best one. The best model included the three main factors, namely block (B), probe dimension (D) and group (G).*
